# Supplementary material for: Engineered ACE2 receptor traps potently neutralize SARS-CoV-2
Source: bioRxiv. 2020 Aug 4:2020.07.31.231746. Originally published 2020 Aug 1. Preprint. [Version 3] doi: 10.1101/2020.07.31.231746 (PMC7402043; doi:10.1101/2020.07.31.231746)
Supplement: Supplement 2 [file media-2.pdf]

Appendix 1: Sequences of ACE2 mutants in this work

13	CAATCTACCATCGAAGAGCAGGCCAAAACATTCCTCGACAAGTTTAATCACGAGGCTGAAGACC<br>TTTTCTACCAATCAAGTCTGGCTAGCTGGAATTACAATACAAACATTACAGAGGAGAACGTACA<br>AAACATGAATAACGCAGGGGACAAGTGGAGCGCATTCCTTAAGGAACAAAGTACCCTTGCGCAA<br>ATGTATCCGCTGCAAGAGATTCAAAACCTGACGGTTAAGCTGCAACTTCAGGCCCTCCAACAAA<br>ATGGAAGTTCAGTCTTGTCAGAAGACAAAAGCAAGCGACTGAACACCATCCTTAACACCATGTC<br>AACCATATATTCAACAGGTAAAGTTTGCAATCCGGATAACCCCCAAGAATGTTTGCTTCTTGAA<br>CCCGGTCTCAACGAAATTATGGCCAACAGTCTTGATTACAACGAGCGATTGTGGGCATGGGAAA<br>GTTGGAGGAGTGAGGTAGGCAAACAGTTGAGACCTCTTTATGAAGAGTACGTTGTCCTTAAAAA<br>TGAAATGGCTCGCGCGAATCATTATGAAGACTATGGTGACTACTGGAGGGGGGATTATGAGGTG<br>AACGGGGTGGACGGATACGATTACTCTAGGGGCCAGCTGATAGAGGATGTCGAGCACACCTTTG<br>AGGAGATTAAGCCGTTGTACGAACATTTGCACGCCTATGTCAGGGCTAAGCTCATGAACGCTTA<br>TCCGAGTTATATCTCCCCGATAGGATGCTTGCCTGCTCACTTGTTGGGCGATATGTGGGGACGC<br>TTTTGGACCAACTTGTATTCCCTTACGGTACCGTTCGGCCAGAAACCAAATATCGACGTGACAG<br>ACGCAATGGTGGATCAAGCATGGGATGCGCAACGAATCTTCAAGGAGGCAGAAAAATTTTTCGT<br>TTCAGTTGGACTCCCAAACATGACGCAGGGTTTCTGGGAGAACTCAATGTTGACAGATCCAGGT<br>AATGTGCAGAAAGCGGTTTGCCACCCTACTGCATGGGATCTTGGTAAAGGGGACTTCCGCATAC<br>TCATGTGTACGAAAGTAACTATGGACGACTTTCTTACTGCGCACCACGAGATGGGGCACATACA<br>ATACGATATGGCGTACGCAGCTCAACCTTTCCTTCTGCGGAACGGGGCGAATGAAGGATTTCAC<br>GAGGCAGTGGGTGAGATTATGTCCCTGTCAGCTGCCACTCCGAAACATCTGAAAAGCATCGGCC<br>TGTTGAGCCCAGACTTCCAAGAAGATAATGAGACCGAAATAAACTTCCTTCTGAAGCAAGCACT<br>GACTATTGTAGGTACCTTGCCCTTTACCTACATGCTGGAGAAGTGGAGGTGGATGGTATTTAAG<br>GGGGAGATACCGAAAGATCAATGGATGAAAAAGTGGTGGGAAATGAAAAGGGAGATCGTTGGCG<br>TAGTTGAACCAGTACCGCATGATGAGACGTACTGCGATCCGGCTAGTCTGTTCCATGTCTCTAA<br>TGATTACTCTTTCATCCGCTACTACACCCGCACGCTGTATCAATTCCAGTTCCAAGAAGCTCTC<br>TGTCAGGCTGCCAAGCACGAAGGACCGCTGCACAAATGCGACATTAGCAATTCTACAGAGGCGG<br>GTCAGAAGTTGTTCAATATGCTTAGACTGGGGAAGAGCGAACCGTGGACGCTCGCTTTGGAGAA<br>CGTTGTTGGAGCTAAGAATATGAACGTCAGGCCCTTGCTGAATTACTTTGAACCTCTGTTTACG<br>TGGTTGAAAGACCAAAATAAAAACTCCTTTGTTGGGTGGAGTACTGACTGGTCCCCCTATGCG
208	CAATCTACCATCGAAGAGCAGGCCAAAACATTCCTCGACAAGTTTAATCACGAGGCTGAAGACC<br>TTTTCTACCAATCAAGTCTGGCTAGCTGGAATTACAATACAAACATTACAGAGGAGAACGTACA<br>AAACATGAATAACGCAGGGGACAAGTGGAGCGCATTCCTTAAGGAACAAAGTACCCTTGCGCAA<br>ATGTATCCGCTGCAAGAGATTCAAAACCTGACGGTTAAGCTGCAACTTCAGGCCCTCCAACAAA<br>ATGGAAGTTCAGTCTTGTCAGAAGACAAAAGCAAGCGACTGAACACCATCCTTAACACCATGTC<br>AACCATATATTCAACAGGTAAAGTTTGCAATCCGGATAACCCCCAAGAATGTTTGCTTCTTGAA<br>CCCGGTCTCAACGAAATTATGGCCAACAGTCTTGATTACAACGAGCGATTGTGGGCATGGGAAA<br>GTTGGAGGAGTGAGGTAGGCAAACAGTTGAGACCTCTTTATGAAGAGTACGTTGTCCTTAAAAA<br>TGAAATGGCTCGCGCGAATCATTATGAAGACTATGGTGACTACTGGAGGGGGGATTATGAGGTG<br>AACGGGGTGGACGGATACGATTACTCTAGGGGCCAGCTGATAGAGGATGTCGAGCACACCTTTG<br>AGGAGATTAAGCCGTTGTACGAACATTTGCACGCCTATGTCAGGGCTAAGCTCATGAACGCTTA<br>TCCGAGTTATATCTCCCCGATAGGATGCTTGCCTGCTCACTTGTTGGGCGATATGTGGGGACGC<br>TTTTGGACCAACTTGTATTCCCTTACGGTACCGTTCGGCCAGAAACCAAATATCGACGTGACAG<br>ACGCAATGGTGGATCAAGCATGGGATGCGCAACGAATCTTCAAGGAGGCAGAAAAATTTTTCGT<br>TTCAGTTGGACTCCCAAACATGACGCAGGGTTTCTGGGAGAACTCAATGTTGACAGATCCAGGT<br>AATGTGCAGAAAGCGGTTTGCCACCCTACTGCATGGGATCTTGGTAAAGGGGACTTCCGCATAC<br>TCATGTGTACGAAAGTAACTATGGACGACTTTCTTACTGCGCACCACGAGATGGGGCACATACA<br>ATACGATATGGCGTACGCAGCTCAACCTTTCCTTCTGCGGAACGGGGCGAATGAAGGATTTCAC

	GAGGCAGTGGGTGAGATTATGTCCCTGTCAGCTGCCACTCCGAAACATCTGAAAAGCATCGGCC<br>TGTTGAGCCCAGACTTCCAAGAAGATAATGAGACCGAAATAAACTTCCTTCTGAAGCAAGCACT<br>GACTATTGTAGGTACCTTGCCCTTTACATACATGCTGGAGAAGTGGAGGTGGATGGTATTTAAG<br>GGGGAGATACCGAAAGATCAATGGATGAAAAAGTGGTGGGAAATGAAAAGGGAGATCGTTGGCG<br>TAGTTGAACCAGTACCGCATGATGAGACGTACTGCGATCCGGCTAGTCTGTTCCATGTCTCTAA<br>TGATTACTCTTTCATCCGCTACTACACCCGCACGCTGTATCAATTCCAGTTCCAAGAAGCTCTC<br>TGTCAGGCTGCCAAGCACGAAGGACCGCTGCACAAATGCGACATTAGCAATTCTACAGAGGCGG<br>GTCAGAAGTTGTTCAATATGCTTAGACTGGGGAAGAGCGAACCGTGGACGCTCGCTTTGGAGAA<br>CGTTGTTGGAGCTAAGAATATGAACGTCAGGCCCTTGCTGAATTACTTTGAACCTCTGTTTACG<br>TGGTTGAAAGACCAAAATAAAAACTCCTTTGTTGGGTGGAGTACTGACTGGTCCCCCTATGCGG<br>ACCAAAGCATCAAAGTGAGGATAAGCCTAAAATCAGCTCTTGGAGATAAAGCATATGAATGGAA<br>CGACAATGAAATGTACCTGTTCCGATCATCTGTTGCATATGCTATGAGGCAGTACTTTTTAAAA<br>GTAAAAAATCAGATGATTCTTTTTGGGGAGGAGGATGTGCGAGTGGCTAATTTGAAACCAAGAA<br>TCTCCTTTAATTTCTTTGTCACTGCACCTAAAAATGTGTCTGATATCATTCCTAGAACTGAAGT<br>TGAAAAGGCCATCAGGATGTCCCGGAGCCGTATCAATGATGCTTTCCGTCTGAATGACAACAGC<br>CTAGAGTTTCTGGGGATACAGCCAACACTTGGACCTCCTAACCAGCCCCCTGTTTCC
Y208	CAATCTACCATCGAAGAGCAGGCCAAAACATTCCTCGACAAGTTTAATCACGAGGCTGAAGACC<br>TTTTCTACCAATCAAGTCTGGCTAGCTGGAATTACAATACAAACATTACAGAGGAGAACGTACA<br>AAACATGAATAACGCAGGGGACAAGTGGAGCGCATTCCTTAAGGAACAAAGTACCCTTGCGCAA<br>ATGTATCCGCTGCAAGAGATTCAAAACCTGACGGTTAAGCTGCAACTTCAGGCCCTCCAACAAA<br>ATGGATCCTCAGTCTTGTCAGAAGACAAAAGCAAGCGACTGAACACCATCCTTAACACCATGTC<br>AACCATATATTCAACAGGTAAAGTTTGCAATCCGGATAACCCCCAAGAATGTTTGCTTCTTGAA<br>CCCGGTCTCAACGAAATTATGGCCAACAGTCTTGATTACAACGAGCGATTGTGGGCATGGGAAA<br>GTTGGAGGAGTGAGGTAGGCAAACAGTTGAGACCTCTTTATGAAGAGTACGTTGTCCTTAAAAA<br>TGAAATGGCTCGCGCGAATCATTATGAAGACTATGGTGACTACTGGAGGGGGGATTATGAGGTG<br>AACGGGGTGGACGGATACGATTACTCTAGGGGCCAGCTGATAGAGGATGTCGAGCACACCTTTG<br>AGGAGATTAAGCCGTTGTACGAACATTTGCACGCCTATGTCAGGGCTAAGCTCATGAACGCTTA<br>TCCGAGTTATATCTCCCCGATAGGATGCTTGCCTGCTCACTTGTTGGGCGATATGTGGGGACGC<br>TTTTGGACCAACTTGTATTCCCTTACGGTACCGTTCGGCCAGAAACCAAATATCGACGTGACAG<br>ACGCAATGGTGGATCAAGCATGGGATGCGCAACGAATCTTCAAGGAGGCAGAAAAATTTTTCGT<br>TTCAGTTGGACTCCCAAACATGACGCAGGGTTTCTGGGAGAACTCAATGTTGACAGATCCAGGT<br>AATGTGCAGAAAGCGGTTTGCCACCCTACTGCATGGGATCTTGGTAAAGGGGACTTCCGCATAC<br>TCATGTGTACGAAAGTAACTATGGACGACTTTCTTACTGCGCACCACGAGATGGGGCACATACA<br>ATACGATATGGCGTACGCAGCTCAACCTTTCCTTCTGCGGAACGGGGCGAATGAAGGATTTCAC<br>GAGGCAGTGGGTGAGATTATGTCCCTGTCAGCTGCCACTCCGAAACATCTGAAAAGCATCGGCC<br>TGTTGAGCCCAGACTTCCAAGAAGATAATGAGACCGAAATAAACTTCCTTCTGAAGCAAGCACT<br>GACTATTGTAGGTACCTTGCCCTTTACATACATGCTGGAGAAGTGGAGGTGGATGGTATTTAAG<br>GGGGAGATACCGAAAGATCAATGGATGAAAAAGTGGTGGGAAATGAAAAGGGAGATCGTTGGCG<br>TAGTTGAACCAGTACCGCATGATGAGACGTACTGCGATCCGGCTAGTCTGTTCCATGTCTCTAA<br>TGATTACTCTTTCATCCGCTACTACACCCGCACGCTGTATCAATTCCAGTTCCAAGAAGCTCTC<br>TGTCAGGCTGCCAAGCACGAAGGACCGCTGCACAAATGCGACATTAGCAATTCTACAGAGGCGG<br>GTCAGAAGTTGTTCAATATGCTTAGACTGGGGAAGAGCGAACCGTGGACGCTCGCTTTGGAGAA<br>CGTTGTTGGAGCTAAGAATATGAACGTCAGGCCCTTGCTGAATTACTTTGAACCTCTGTTTACG<br>TGGTTGAAAGACCAAAATAAAAACTCCTTTGTTGGGTGGAGTACTGACTGGTCCCCCTATGCG
14	CAATCTACCATCGAAGAGCAGGCCAAAACATTCCTCGACAAGTTTAATGTCGAGGCTGAAGACC<br>TTTTCTACCAATCAAGTCTGGCTAGCTGGAATTACAATACAAACATTACAGAGGAGAACGTACA<br>AAACATGAATAACGCAGGGGACAAGTGGAGCGCATTCCTTAAGGAACAAAGTACCCTTGCGCAA

	ATGTATCCGCTGCAAGAGATTCAAAACCTGACGGTTAAGCTGCAACTTCAGGCCCTCCAACAAA<br>ATGGAAGTTCAGTCTTGTCAGAAGACAAAAGCAAGCGACTGAACACCATCCTTAACACCATGTC<br>AACCATATATTCAACAGGTAAAGTTTGCAATCCGGATAACCCCCAAGAATGTTTGCTTCTTGAA<br>CCCGGTCTCAACGAAATTATGGCCAACAGTCTTGATTACAACGAGCGATTGTGGGCATGGGAAA<br>GTTGGAGGAGTGAGGTAGGCAAACAGTTGAGACCTCTTTATGAAGAGTACGTTGTCCTTAAAAA<br>TGAAATGGCTCGCGCGAATCATTATGAAGACTATGGTGACTACTGGAGGGGGGATTATGAGGTG<br>AACGGGGTGGACGGATACGATTACTCTAGGGGCCAGCTGATAGAGGATGTCGAGCACACCTTTG<br>AGGAGATTAAGCCGTTGTACGAACATTTGCACGCCTATGTCAGGGCTAAGCTCATGAACGCTTA<br>TCCGAGTTATATCTCCCCGATAGGATGCTTGCCTGCTCACTTGTTGGGCGATATGTGGGGACGC<br>TTTTGGACCAACTTGTATTCCCTTACGGTACCGTTCGGCCAGAAACCAAATATCGACGTGACAG<br>ACGCAATGGTGGATCAAGCATGGGATGCGCAACGAATCTTCAAGGAGGCAGAAAAATTTTTCGT<br>TTCAGTTGGACTCCCAAACATGACGCAGGGTTTCTGGGAGAACTCAATGTTGACAGATCCAGGT<br>AATGTGCAGAAAGCGGTTTGCCACCCTACTGCATGGGATCTTGGTAAAGGGGACTTCCGCATAC<br>TCATGTGTACGAAAGTAACTATGGACGACTTTCTTACTGCGCACCACGAGATGGGGCACATACA<br>ATACGATATGGCGTACGCAGCTCAACCTTTCCTTCTGCGGAACGGGGCGAATGAAGGATTTCAC<br>GAGGCAGTGGGTGAGATTATGTCCCTGTCAGCTGCCACTCCGAAACATCTGAAAAGCATCGGCC<br>TGTTGAGCCCAGACTTCCAAGAAGATAATGAGACCGAAATAAACTTCCTTCTGAAGCAAGCACT<br>GACTATTGTAGGTACCTTGCCCTTTACCTACATGCTGGAGAAGTGGAGGTGGATGGTATTTAAG<br>GGGGAGATACCGAAAGATCAATGGATGAAAAAGTGGTGGGAAATGAAAAGGGAGATCGTTGGCG<br>TAGTTGAACCAGTACCGCATGATGAGACGTACTGCGATCCGGCTAGTCTGTTCCATGTCTCTAA<br>TGATTACTCTTTCATCCGCTACTACACCCGCACGCTGTATCAATTCCAGTTCCAAGAAGCTCTC<br>TGTCAGGCTGCCAAGCACGAAGGACCGCTGCACAAATGCGACATTAGCAATTCTACAGAGGCGG<br>GTCAGAAGTTGTTCAATATGCTTAGACTGGGGAAGAGCGAACCGTGGACGCTCGCTTTGGAGAA<br>CGTTGTTGGAGCTAAGAATATGAACGTCAGGCCCTTGCTGAATTACTTTGAACCTCTGTTTACG<br>TGGTTGAAAGACCAAAATAAAAACTCCTTTGTTGGGTGGAGTACTGACTGGTCCCCCTATGCG
295	CAATCTACCATCGAAGAGCAGGCCAAAACATTCCTCGACAAGTTTAATGTCGAGGCTGAAGACC<br>TTTTCTACCAATCAAGTCTGGCTAGCTGGAATTACAATACAAACATTACAGAGGAGAACGTACA<br>AAACATGAATAACGCAGGGGACAAGTGGAGCGCATTCCTTAAGGAACAAAGTACCCTTGCGCAA<br>ATGTATCCGCTGCAAGAGATTCAAAACCTGACGGTTAAGCTGCAACTTCAGGCCCTCCAACAAA<br>ATGGATCCTCAGTCTTGTCAGAAGACAAAAGCAAGCGACTGAACACCATCCTTAACACCATGTC<br>AACCATATATTCAACAGGTAAAGTTTGCAATCCGGATAACCCCCAAGAATGTTTGCTTCTTGAA<br>CCCGGTCTCAACGAAATTATGGCCAACAGTCTTGATTACAACGAGCGATTGTGGGCATGGGAAA<br>GTTGGAGGAGTGAGGTAGGCAAACAGTTGAGACCTCTTTATGAAGAGTACGTTGTCCTTAAAAA<br>TGAAATGGCTCGCGCGAATCATTATGAAGACTATGGTGACTACTGGAGGGGGGATTATGAGGTG<br>AACGGGGTGGACGGATACGATTACTCTAGGGGCCAGCTGATAGAGGATGTCGAGCACACCTTTG<br>AGGAGATTAAGCCGTTGTACGAACATTTGCACGCCTATGTCAGGGCTAAGCTCATGAACGCTTA<br>TCCGAGTTATATCTCCCCGATAGGATGCTTGCCTGCTCACTTGTTGGGCGATATGTGGGGACGC<br>TTTTGGACCAACTTGTATTCCCTTACGGTACCGTTCGGCCAGAAACCAAATATCGACGTGACAG<br>ACGCAATGGTGGATCAAGCATGGGATGCGCAACGAATCTTCAAGGAGGCAGAAAAATTTTTCGT<br>TTCAGTTGGACTCCCAAACATGACGCAGGGTTTCTGGGAGAACTCAATGTTGACAGATCCAGGT<br>AATGTGCAGAAAGCGGTTTGCCACCCTACTGCATGGGATCTTGGTAAAGGGGACTTCCGCATAC<br>TCATGTGTACGAAAGTAACTATGGACGACTTTCTTACTGCGCACCACGAGATGGGGCACATACA<br>ATACGATATGGCGTACGCAGCTCAACCTTTCCTTCTGCGGAACGGGGCGAATGAAGGATTTCAC<br>GAGGCAGTGGGTGAGATTATGTCCCTGTCAGCTGCCACTCCGAAACATCTGAAAAGCATCGGCC<br>TGTTGAGCCCAGACTTCCAAGAAGATAATGAGACCGAAATAAACTTCCTTCTGAAGCAAGCACT<br>GACTATTGTAGGTACCTTGCCCTTTACATACATGCTGGAGAAGTGGAGGTGGATGGTATTTAAG<br>GGGGAGATACCGAAAGATCAATGGATGAAAAAGTGGTGGGAAATGAAAAGGGAGATCGTTGGCG

	TAGTTGAACCAGTACCGCATGATGAGACGTACTGCGATCCGGCTAGTCTGTTCCATGTCTCTAA
	TGATTACTCTTTCATCCGCTACTACACCCGCACGCTGTATCAATTCCAGTTCCAAGAAGCTCTC
	TGTCAGGCTGCCAAGCACGAAGGACCGCTGCACAAATGCGACATTAGCAATTCTACAGAGGCGG
	GTCAGAAGTTGTTCAATATGCTTAGACTGGGGAAGAGCGAACCGTGGACGCTCGCTTTGGAGAA
	CGTTGTTGGAGCTAAGAATATGAACGTCAGGCCCTTGCTGAATTACTTTGAACCTCTGTTTACG
	TGGTTGAAAGACCAAAATAAAAACTCCTTTGTTGGGTGGAGTACTGACTGGTCCCCCTATGCGG
	ACCAAAGCATCAAAGTGAGGATAAGCCTAAAATCAGCTCTTGGAGATAAAGCATATGAATGGAA
	CGACAATGAAATGTACCTGTTCCGATCATCTGTTGCATATGCTATGAGGCAGTACTTTTTAAAA
	GTAAAAAATCAGATGATTCTTTTTGGGGAGGAGGATGTGCGAGTGGCTAATTTGAAACCAAGAA
	TCTCCTTTAATTTCTTTGTCACTGCACCTAAAAATGTGTCTGATATCATTCCTAGAACTGAAGT
	TGAAAAGGCCATCAGGATGTCCCGGAGCCGTATCAATGATGCTTTCCGTCTGAATGACAACAGC
	CTAGAGTTTCTGGGGATACAGCCAACACTTGGACCTCCTAACCAGCCCCCTGTTTCC
19	CAATCTACCATCGAAGAGCAGGCCAAAACATTCCTCGACTTCTTTAATATCCAGGCTGAAGACC
	TTTTCTACCAATCAAGTCTGGCTAGCTGGAATTACAATACAAACATTACAGAGGAGAACGTACA
	AAACATGAATAACGCAGGGGACAAGTGGAGCGCATTCCTTAAGGAACAAAGTACCCTTGCGCAA
	ATGTATCCGCTGCAAGAGATTCAAAACCTGACGGTTAAGCTGCAACTTCAGGCCCTCCAACAAA
	ATGGAAGTTCAGTCTTGTCAGAAGACAAAAGCAAGCGACTGAACACCATCCTTAACACCATGTC
	AACCATATATTCAACAGGTAAAGTTTGCAATCCGGATAACCCCCAAGAATGTTTGCTTCTTGAA
	CCCGGTCTCAACGAAATTATGGCCAACAGTCTTGATTACAACGAGCGATTGTGGGCATGGGAAA
	GTTGGAGGAGTGAGGTAGGCAAACAGTTGAGACCTCTTTATGAAGAGTACGTTGTCCTTAAAAA
	TGAAATGGCTCGCGCGAATCATTATGAAGACTATGGTGACTACTGGAGGGGGGATTATGAGGTG
	AACGGGGTGGACGGATACGATTACTCTAGGGGCCAGCTGATAGAGGATGTCGAGCACACCTTTG
	AGGAGATTAAGCCGTTGTACGAACATTTGCACGCCTATGTCAGGGCTAAGCTCATGAACGCTTA
	TCCGAGTTATATCTCCCCGATAGGATGCTTGCCTGCTCACTTGTTGGGCGATATGTGGGGACGC
	TTTTGGACCAACTTGTATTCCCTTACGGTACCGTTCGGCCAGAAACCAAATATCGACGTGACAG
	ACGCAATGGTGGATCAAGCATGGGATGCGCAACGAATCTTCAAGGAGGCAGAAAAATTTTTCGT
	TTCAGTTGGACTCCCAAACATGACGCAGGGTTTCTGGGAGAACTCAATGTTGACAGATCCAGGT
	AATGTGCAGAAAGCGGTTTGCCACCCTACTGCATGGGATCTTGGTAAAGGGGACTTCCGCATAC
	TCATGTGTACGAAAGTAACTATGGACGACTTTCTTACTGCGCACCACGAGATGGGGCACATACA
	ATACGATATGGCGTACGCAGCTCAACCTTTCCTTCTGCGGAACGGGGCGAATGAAGGATTTCAC
	GAGGCAGTGGGTGAGATTATGTCCCTGTCAGCTGCCACTCCGAAACATCTGAAAAGCATCGGCC
	TGTTGAGCCCAGACTTCCAAGAAGATAATGAGACCGAAATAAACTTCCTTCTGAAGCAAGCACT
	GACTATTGTAGGTACCTTGCCCTTTACCTACATGCTGGAGAAGTGGAGGTGGATGGTATTTAAG
	GGGGAGATACCGAAAGATCAATGGATGAAAAAGTGGTGGGAAATGAAAAGGGAGATCGTTGGCG
	TAGTTGAACCAGTACCGCATGATGAGACGTACTGCGATCCGGCTAGTCTGTTCCATGTCTCTAA
	TGATTACTCTTTCATCCGCTACTACACCCGCACGCTGTATCAATTCCAGTTCCAAGAAGCTCTC
	TGTCAGGCTGCCAAGCACGAAGGACCGCTGCACAAATGCGACATTAGCAATTCTACAGAGGCGG
	GTCAGAAGTTGTTCAATATGCTTAGACTGGGGAAGAGCGAACCGTGGACGCTCGCTTTGGAGAA
	CGTTGTTGGAGCTAAGAATATGAACGTCAGGCCCTTGCTGAATTACTTTGAACCTCTGTTTACG
	TGGTTGAAAGACCAAAATAAAAACTCCTTTGTTGGGTGGAGTACTGACTGGTCCCCCTATGCG
293	CAATCTACCATCGAAGAGCAGGCCAAAACATTCCTCGACTTCTTTAATATCCAGGCTGAAGACC
	TTTTCTACCAATCAAGTCTGGCTAGCTGGAATTACAATACAAACATTACAGAGGAGAACGTACA
	AAACATGAATAACGCAGGGGACAAGTGGAGCGCATTCCTTAAGGAACAAAGTACCCTTGCGCAA
	ATGTATCCGCTGCAAGAGATTCAAAACCTGACGGTTAAGCTGCAACTTCAGGCCCTCCAACAAA
	ATGGAAGTTCAGTCTTGTCAGAAGACAAAAGCAAGCGACTGAACACCATCCTTAACACCATGTC
	AACCATATATTCAACAGGTAAAGTTTGCAATCCGGATAACCCCCAAGAATGTTTGCTTCTTGAA
	CCCGGTCTCAACGAAATTATGGCCAACAGTCTTGATTACAACGAGCGATTGTGGGCATGGGAAA

	GTTGGAGGAGTGAGGTAGGCAAACAGTTGAGACCTCTTTATGAAGAGTACGTTGTCCTTAAAAA<br>TGAAATGGCTCGCGCGAATCATTATGAAGACTATGGTGACTACTGGAGGGGGGATTATGAGGTG<br>AACGGGGTGGACGGATACGATTACTCTAGGGGCCAGCTGATAGAGGATGTCGAGCACACCTTTG<br>AGGAGATTAAGCCGTTGTACGAACATTTGCACGCCTATGTCAGGGCTAAGCTCATGAACGCTTA<br>TCCGAGTTATATCTCCCCGATAGGATGCTTGCCTGCTCACTTGTTGGGCGATATGTGGGGACGC<br>TTTTGGACCAACTTGTATTCCCTTACGGTACCGTTCGGCCAGAAACCAAATATCGACGTGACAG<br>ACGCAATGGTGGATCAAGCATGGGATGCGCAACGAATCTTCAAGGAGGCAGAAAAATTTTTCGT<br>TTCAGTTGGACTCCCAAACATGACGCAGGGTTTCTGGGAGAACTCAATGTTGACAGATCCAGGT<br>AATGTGCAGAAAGCGGTTTGCCACCCTACTGCATGGGATCTTGGTAAAGGGGACTTCCGCATAC<br>TCATGTGTACGAAAGTAACTATGGACGACTTTCTTACTGCGCACCACGAGATGGGGCACATACA<br>ATACGATATGGCGTACGCAGCTCAACCTTTCCTTCTGCGGAACGGGGCGAATGAAGGATTTCAC<br>GAGGCAGTGGGTGAGATTATGTCCCTGTCAGCTGCCACTCCGAAACATCTGAAAAGCATCGGCC<br>TGTTGAGCCCAGACTTCCAAGAAGATAATGAGACCGAAATAAACTTCCTTCTGAAGCAAGCACT<br>GACTATTGTAGGTACCTTGCCCTTTACATACATGCTGGAGAAGTGGAGGTGGATGGTATTTAAG<br>GGGGAGATACCGAAAGATCAATGGATGAAAAAGTGGTGGGAAATGAAAAGGGAGATCGTTGGCG<br>TAGTTGAACCAGTACCGCATGATGAGACGTACTGCGATCCGGCTAGTCTGTTCCATGTCTCTAA<br>TGATTACTCTTTCATCCGCTACTACACCCGCACGCTGTATCAATTCCAGTTCCAAGAAGCTCTC<br>TGTCAGGCTGCCAAGCACGAAGGACCGCTGCACAAATGCGACATTAGCAATTCTACAGAGGCGG<br>GTCAGAAGTTGTTCAATATGCTTAGACTGGGGAAGAGCGAACCGTGGACGCTCGCTTTGGAGAA<br>CGTTGTTGGAGCTAAGAATATGAACGTCAGGCCCTTGCTGAATTACTTTGAACCTCTGTTTACG<br>TGGTTGAAAGACCAAAATAAAAACTCCTTTGTTGGGTGGAGTACTGACTGGTCCCCCTATGCGG<br>ACCAAAGCATCAAAGTGAGGATAAGCCTAAAATCAGCTCTTGGAGATAAAGCATATGAATGGAA<br>CGACAATGAAATGTACCTGTTCCGATCATCTGTTGCATATGCTATGAGGCAGTACTTTTTAAAA<br>GTAAAAAATCAGATGATTCTTTTTGGGGAGGAGGATGTGCGAGTGGCTAATTTGAAACCAAGAA<br>TCTCCTTTAATTTCTTTGTCACTGCACCTAAAAATGTGTCTGATATCATTCCTAGAACTGAAGT<br>TGAAAAGGCCATCAGGATGTCCCGGAGCCGTATCAATGATGCTTTCCGTCTGAATGACAACAGC<br>CTAGAGTTTCTGGGGATACAGCCAACACTTGGACCTCCTAACCAGCCCCCTGTTTCC
117	CAATCTACCATCGAAGAGCAGGCCAAAACATTCCTCGACAAGTTTAATCACGAGGCTGAAGACC<br>TTTTCTACCAATCAAGTCTGGCTAGCTGGAATTACAATACAAACATTACAGAGGAGAACGTACA<br>AAACATGAATAACGCAGGGGACAAGTGGAGCGCATTCCTTAAGGAACAAAGTACCCTTGCGCAA<br>ATGTATCCGCTGCAAGAGATTCAACAACTGACGGTTAAGCTGCAACTTCAGGCCCTCCAACAAA<br>ATGGATCCTCAGTCTTGTCAGAAGACAAAAGCAAGCGACTGAACACCATCCTTAACACCATGTC<br>AACCATATATTCAACAGGTAAAGTTTGCAATCCGGATAACCCCCAAGAATGTTTGCTTCTTGAA<br>CCCGGTCTCAACGAAATTATGGCCAACAGTCTTGATTACAACGAGCGATTGTGGGCATGGGAAA<br>GTTGGAGGAGTGAGGTAGGCAAACAGTTGAGACCTCTTTATGAAGAGTACGTTGTCCTTAAAAA<br>TGAAATGGCTCGCGCGAATCATTATGAAGACTATGGTGACTACTGGAGGGGGGATTATGAGGTG<br>AACGGGGTGGACGGATACGATTACTCTAGGGGCCAGCTGATAGAGGATGTCGAGCACACCTTTG<br>AGGAGATTAAGCCGTTGTACGAACATTTGCACGCCTATGTCAGGGCTAAGCTCATGAACGCTTA<br>TCCGAGTTATATCTCCCCGATAGGATGCTTGCCTGCTCACTTGTTGGGCGATATGTGGGGACGC<br>TTTTGGACCAACTTGTATTCCCTTACGGTACCGTTCGGCCAGAAACCAAATATCGACGTGACAG<br>ACGCAATGGTGGATCAAGCATGGGATGCGCAACGAATCTTCAAGGAGGCAGAAAAATTTTTCGT<br>TTCAGTTGGACTCCCAAACATGACGCAGGGTTTCTGGGAGAACTCAATGTTGACAGATCCAGGT<br>AATGTGCAGAAAGCGGTTTGCCACCCTACTGCATGGGATCTTGGTAAAGGGGACTTCCGCATAC<br>TCATGTGTACGAAAGTAACTATGGACGACTTTCTTACTGCGCACAACGAGATGGGGAACATACA<br>ATACGATATGGCGTACGCAGCTCAACCTTTCCTTCTGCGGAACGGGGCGAATGAAGGATTTCAC<br>GAGGCAGTGGGTGAGATTATGTCCCTGTCAGCTGCCACTCCGAAACATCTGAAAAGCATCGGCC<br>TGTTGAGCCCAGACTTCCAAGAAGATAATGAGACCGAAATAAACTTCCTTCTGAAGCAAGCACT

	GACTATTGTAGGTACCTTGCCCTTTACATACATGCTGGAGAAGTGGAGGTGGATGGTATTTAAG<br>GGGGAGATACCGAAAGATCAATGGATGAAAAAGTGGTGGGAAATGAAAAGGGAGATCGTTGGCG<br>TAGTTGAACCAGTACCGCATGATGAGACGTACTGCGATCCGGCTAGTCTGTTCCATGTCTCTAA<br>TGATTACTCTTTCATCCGCTACTACACCCGCACGCTGTATCAATTCCAGTTCCAAGAAGCTCTC<br>TGTCAGGCTGCCAAGCACGAAGGACCGCTGCACAAATGCGACATTAGCAATTCTACAGAGGCGG<br>GTCAGAAGTTGTTCAATATGCTTAGACTGGGGAAGAGCGAACCGTGGACGCTCGCTTTGGAGAA<br>CGTTGTTGGAGCTAAGAATATGAACGTCAGGCCCTTGCTGAATTACTTTGAACCTCTGTTTACG<br>TGGTTGAAAGACCAAAATAAAAACTCCTTTGTTGGGTGGAGTACTGACTGGTCCCCCTATGCG
Y117	CAATCTACCATCGAAGAGCAGGCCAAAACATTCCTCGACAAGTTTAATCACGAGGCTGAAGACC<br>TTTTCTACCAATCAAGTCTGGCTAGCTGGAATTACAATACAAACATTACAGAGGAGAACGTACA<br>AAACATGAATAACGCAGGGGACAAGTGGAGCGCATTCCTTAAGGAACAAAGTACCCTTGCGCAA<br>ATGTATCCGCTGCAAGAGATTCAACAACTGACGGTTAAGCTGCAACTTCAGGCCCTCCAACAAA<br>ATGGATCCTCAGTCTTGTCAGAAGACAAAAGCAAGCGACTGAACACCATCCTTAACACCATGTC<br>AACCATATATTCAACAGGTAAAGTTTGCAATCCGGATAACCCCCAAGAATGTTTGCTTCTTGAA<br>CCCGGTCTCAACGAAATTATGGCCAACAGTCTTGATTACAACGAGCGATTGTGGGCATGGGAAA<br>GTTGGAGGAGTGAGGTAGGCAAACAGTTGAGACCTCTTTATGAAGAGTACGTTGTCCTTAAAAA<br>TGAAATGGCTCGCGCGAATCATTATGAAGACTATGGTGACTACTGGAGGGGGGATTATGAGGTG<br>AACGGGGTGGACGGATACGATTACTCTAGGGGCCAGCTGATAGAGGATGTCGAGCACACCTTTG<br>AGGAGATTAAGCCGTTGTACGAACATTTGCACGCCTATGTCAGGGCTAAGCTCATGAACGCTTA<br>TCCGAGTTATATCTCCCCGATAGGATGCTTGCCTGCTCACTTGTTGGGCGATATGTGGGGACGC<br>TTTTGGACCAACTTGTATTCCCTTACGGTACCGTTCGGCCAGAAACCAAATATCGACGTGACAG<br>ACGCAATGGTGGATCAAGCATGGGATGCGCAACGAATCTTCAAGGAGGCAGAAAAATTTTTCGT<br>TTCAGTTGGACTCCCAAACATGACGCAGGGTTTCTGGGAGAACTCAATGTTGACAGATCCAGGT<br>AATGTGCAGAAAGCGGTTTGCCACCCTACTGCATGGGATCTTGGTAAAGGGGACTTCCGCATAC<br>TCATGTGTACGAAAGTAACTATGGACGACTTTCTTACTGCGCACCACGAGATGGGGCACATACA<br>ATACGATATGGCGTACGCAGCTCAACCTTTCCTTCTGCGGAACGGGGCGAATGAAGGATTTCAC<br>GAGGCAGTGGGTGAGATTATGTCCCTGTCAGCTGCCACTCCGAAACATCTGAAAAGCATCGGCC<br>TGTTGAGCCCAGACTTCCAAGAAGATAATGAGACCGAAATAAACTTCCTTCTGAAGCAAGCACT<br>GACTATTGTAGGTACCTTGCCCTTTACATACATGCTGGAGAAGTGGAGGTGGATGGTATTTAAG<br>GGGGAGATACCGAAAGATCAATGGATGAAAAAGTGGTGGGAAATGAAAAGGGAGATCGTTGGCG<br>TAGTTGAACCAGTACCGCATGATGAGACGTACTGCGATCCGGCTAGTCTGTTCCATGTCTCTAA<br>TGATTACTCTTTCATCCGCTACTACACCCGCACGCTGTATCAATTCCAGTTCCAAGAAGCTCTC<br>TGTCAGGCTGCCAAGCACGAAGGACCGCTGCACAAATGCGACATTAGCAATTCTACAGAGGCGG<br>GTCAGAAGTTGTTCAATATGCTTAGACTGGGGAAGAGCGAACCGTGGACGCTCGCTTTGGAGAA<br>CGTTGTTGGAGCTAAGAATATGAACGTCAGGCCCTTGCTGAATTACTTTGAACCTCTGTTTACG<br>TGGTTGAAAGACCAAAATAAAAACTCCTTTGTTGGGTGGAGTACTGACTGGTCCCCCTATGCG
118	CAATCTACCATCGAAGAGCAGGCCAAAACATTCCTCGACAAGTTTAATGTCGAGGCTGAAGACC<br>TTTTCTACCAATCAAGTCTGGCTAGCTGGAATTACAATACAAACATTACAGAGGAGAACGTACA<br>AAACATGAATAACGCAGGGGACAAGTGGAGCGCATTCCTTAAGGAACAAAGTACCCTTGCGCAA<br>ATGTATCCGCTGCAAGAGATTCAACAACTGACGGTTAAGCTGCAACTTCAGGCCCTCCAACAAA<br>ATGGATCCTCAGTCTTGTCAGAAGACAAAAGCAAGCGACTGAACACCATCCTTAACACCATGTC<br>AACCATATATTCAACAGGTAAAGTTTGCAATCCGGATAACCCCCAAGAATGTTTGCTTCTTGAA<br>CCCGGTCTCAACGAAATTATGGCCAACAGTCTTGATTACAACGAGCGATTGTGGGCATGGGAAA<br>GTTGGAGGAGTGAGGTAGGCAAACAGTTGAGACCTCTTTATGAAGAGTACGTTGTCCTTAAAAA<br>TGAAATGGCTCGCGCGAATCATTATGAAGACTATGGTGACTACTGGAGGGGGGATTATGAGGTG<br>AACGGGGTGGACGGATACGATTACTCTAGGGGCCAGCTGATAGAGGATGTCGAGCACACCTTTG<br>AGGAGATTAAGCCGTTGTACGAACATTTGCACGCCTATGTCAGGGCTAAGCTCATGAACGCTTA

	TCCGAGTTATATCTCCCCGATAGGATGCTTGCCTGCTCACTTGTTGGGCGATATGTGGGGACGC<br>TTTTGGACCAACTTGTATTCCCTTACGGTACCGTTCGGCCAGAAACCAAATATCGACGTGACAG<br>ACGCAATGGTGGATCAAGCATGGGATGCGCAACGAATCTTCAAGGAGGCAGAAAAATTTTTCGT<br>TTCAGTTGGACTCCCAAACATGACGCAGGGTTTCTGGGAGAACTCAATGTTGACAGATCCAGGT<br>AATGTGCAGAAAGCGGTTTGCCACCCTACTGCATGGGATCTTGGTAAAGGGGACTTCCGCATAC<br>TCATGTGTACGAAAGTAACTATGGACGACTTTCTTACTGCGCACAACGAGATGGGGAACATACA<br>ATACGATATGGCGTACGCAGCTCAACCTTTCCTTCTGCGGAACGGGGCGAATGAAGGATTTCAC<br>GAGGCAGTGGGTGAGATTATGTCCCTGTCAGCTGCCACTCCGAAACATCTGAAAAGCATCGGCC<br>TGTTGAGCCCAGACTTCCAAGAAGATAATGAGACCGAAATAAACTTCCTTCTGAAGCAAGCACT<br>GACTATTGTAGGTACCTTGCCCTTTACATACATGCTGGAGAAGTGGAGGTGGATGGTATTTAAG<br>GGGGAGATACCGAAAGATCAATGGATGAAAAAGTGGTGGGAAATGAAAAGGGAGATCGTTGGCG<br>TAGTTGAACCAGTACCGCATGATGAGACGTACTGCGATCCGGCTAGTCTGTTCCATGTCTCTAA<br>TGATTACTCTTTCATCCGCTACTACACCCGCACGCTGTATCAATTCCAGTTCCAAGAAGCTCTC<br>TGTCAGGCTGCCAAGCACGAAGGACCGCTGCACAAATGCGACATTAGCAATTCTACAGAGGCGG<br>GTCAGAAGTTGTTCAATATGCTTAGACTGGGGAAGAGCGAACCGTGGACGCTCGCTTTGGAGAA<br>CGTTGTTGGAGCTAAGAATATGAACGTCAGGCCCTTGCTGAATTACTTTGAACCTCTGTTTACG<br>TGGTTGAAAGACCAAAATAAAAACTCCTTTGTTGGGTGGAGTACTGACTGGTCCCCCTATGCG
278	CAATCTACCATCGAAGAGCAGGCCAAAACATTCCTCGACAAGTTTAATGTCGAGGCTGAAGACC<br>TTTTCTACCAATCAAGTCTGGCTAGCTGGAATTACAATACAAACATTACAGAGGAGAACGTACA<br>AAACATGAATAACGCAGGGGACAAGTGGAGCGCATTCCTTAAGGAACAAAGTACCCTTGCGCAA<br>ATGTATCCGCTGCAAGAGATTCAACAACTGACGGTTAAGCTGCAACTTCAGGCCCTCCAACAAA<br>ATGGATCCTCAGTCTTGTCAGAAGACAAAAGCAAGCGACTGAACACCATCCTTAACACCATGTC<br>AACCATATATTCAACAGGTAAAGTTTGCAATCCGGATAACCCCCAAGAATGTTTGCTTCTTGAA<br>CCCGGTCTCAACGAAATTATGGCCAACAGTCTTGATTACAACGAGCGATTGTGGGCATGGGAAA<br>GTTGGAGGAGTGAGGTAGGCAAACAGTTGAGACCTCTTTATGAAGAGTACGTTGTCCTTAAAAA<br>TGAAATGGCTCGCGCGAATCATTATGAAGACTATGGTGACTACTGGAGGGGGGATTATGAGGTG<br>AACGGGGTGGACGGATACGATTACTCTAGGGGCCAGCTGATAGAGGATGTCGAGCACACCTTTG<br>AGGAGATTAAGCCGTTGTACGAACATTTGCACGCCTATGTCAGGGCTAAGCTCATGAACGCTTA<br>TCCGAGTTATATCTCCCCGATAGGATGCTTGCCTGCTCACTTGTTGGGCGATATGTGGGGACGC<br>TTTTGGACCAACTTGTATTCCCTTACGGTACCGTTCGGCCAGAAACCAAATATCGACGTGACAG<br>ACGCAATGGTGGATCAAGCATGGGATGCGCAACGAATCTTCAAGGAGGCAGAAAAATTTTTCGT<br>TTCAGTTGGACTCCCAAACATGACGCAGGGTTTCTGGGAGAACTCAATGTTGACAGATCCAGGT<br>AATGTGCAGAAAGCGGTTTGCCTCCCTACTGCATGGGATCTTGGTAAAGGGGACTTCCGCATAC<br>TCATGTGTACGAAAGTAACTATGGACGACTTTCTTACTGCGCACCACGAGATGGGGCACATACA<br>ATACGATATGGCGTACGCAGCTCAACCTTTCCTTCTGCGGAACGGGGCGAATGAAGGATTTCAC<br>GAGGCAGTGGGTGAGATTATGTCCCTGTCAGCTGCCACTCCGAAACATCTGAAAAGCATCGGCC<br>TGTTGAGCCCAGACTTCCAAGAAGATAATGAGACCGAAATAAACTTCCTTCTGAAGCAAGCACT<br>GACTATTGTAGGTACCTTGCCCTTTACATACATGCTGGAGAAGTGGAGGTGGATGGTATTTAAG<br>GGGGAGATACCGAAAGATCAATGGATGAAAAAGTGGTGGGAAATGAAAAGGGAGATCGTTGGCG<br>TAGTTGAACCAGTACCGCATGATGAGACGTACTGCGATCCGGCTAGTCTGTTCCATGTCTCTAA<br>TGATTACTCTTTCATCCGCTACTACACCCGCACGCTGTATCAATTCCAGTTCCAAGAAGCTCTC<br>TGTCAGGCTGCCAAGCACGAAGGACCGCTGCACAAATGCGACATTAGCAATTCTACAGAGGCGG<br>GTCAGAAGTTGTTCAATATGCTTAGACTGGGGAAGAGCGAACCGTGGACGCTCGCTTTGGAGAA<br>CGTTGTTGGAGCTAAGAATATGAACGTCAGGCCCTTGCTGAATTACTTTGAACCTCTGTTTACG<br>TGGTTGAAAGACCAAAATAAAAACTCCTTTGTTGGGTGGAGTACTGACTGGTCCCCCTATGCG
292	CAATCTACCATCGAAGAGCAGGCCAAAACATTCCTCGACAAGTTTAATGTCGAGGCTGAAGACC<br>TTTTCTACCAATCAAGTCTGGCTAGCTGGAATTACAATACAAACATTACAGAGGAGAACGTACA

	AAACATGAATAACGCAGGGGACAAGTGGAGCGCATTCCTTAAGGAACAAAGTACCCTTGCGCAA<br>ATGTATCCGCTGCAAGAGATTCAACAACTGACGGTTAAGCTGCAACTTCAGGCCCTCCAACAAA<br>ATGGATCCTCAGTCTTGTCAGAAGACAAAAGCAAGCGACTGAACACCATCCTTAACACCATGTC<br>AACCATATATTCAACAGGTAAAGTTTGCAATCCGGATAACCCCCAAGAATGTTTGCTTCTTGAA<br>CCCGGTCTCAACGAAATTATGGCCAACAGTCTTGATTACAACGAGCGATTGTGGGCATGGGAAA<br>GTTGGAGGAGTGAGGTAGGCAAACAGTTGAGACCTCTTTATGAAGAGTACGTTGTCCTTAAAAA<br>TGAAATGGCTCGCGCGAATCATTATGAAGACTATGGTGACTACTGGAGGGGGGATTATGAGGTG<br>AACGGGGTGGACGGATACGATTACTCTAGGGGCCAGCTGATAGAGGATGTCGAGCACACCTTTG<br>AGGAGATTAAGCCGTTGTACGAACATTTGCACGCCTATGTCAGGGCTAAGCTCATGAACGCTTA<br>TCCGAGTTATATCTCCCCGATAGGATGCTTGCCTGCTCACTTGTTGGGCGATATGTGGGGACGC<br>TTTTGGACCAACTTGTATTCCCTTACGGTACCGTTCGGCCAGAAACCAAATATCGACGTGACAG<br>ACGCAATGGTGGATCAAGCATGGGATGCGCAACGAATCTTCAAGGAGGCAGAAAAATTTTTCGT<br>TTCAGTTGGACTCCCAAACATGACGCAGGGTTTCTGGGAGAACTCAATGTTGACAGATCCAGGT<br>AATGTGCAGAAAGCGGTTTGCCACCCTACTGCATGGGATCTTGGTAAAGGGGACTTCCGCATAC<br>TCATGTGTACGAAAGTAACTATGGACGACTTTCTTACTGCGCACCACGAGATGGGGCACATACA<br>ATACGATATGGCGTACGCAGCTCAACCTTTCCTTCTGCGGAACGGGGCGAATGAAGGATTTCAC<br>GAGGCAGTGGGTGAGATTATGTCCCTGTCAGCTGCCACTCCGAAACATCTGAAAAGCATCGGCC<br>TGTTGAGCCCAGACTTCCAAGAAGATAATGAGACCGAAATAAACTTCCTTCTGAAGCAAGCACT<br>GACTATTGTAGGTACCTTGCCCTTTACATACATGCTGGAGAAGTGGAGGTGGATGGTATTTAAG<br>GGGGAGATACCGAAAGATCAATGGATGAAAAAGTGGTGGGAAATGAAAAGGGAGATCGTTGGCG<br>TAGTTGAACCAGTACCGCATGATGAGACGTACTGCGATCCGGCTAGTCTGTTCCATGTCTCTAA<br>TGATTACTCTTTCATCCGCTACTACACCCGCACGCTGTATCAATTCCAGTTCCAAGAAGCTCTC<br>TGTCAGGCTGCCAAGCACGAAGGACCGCTGCACAAATGCGACATTAGCAATTCTACAGAGGCGG<br>GTCAGAAGTTGTTCAATATGCTTAGACTGGGGAAGAGCGAACCGTGGACGCTCGCTTTGGAGAA<br>CGTTGTTGGAGCTAAGAATATGAACGTCAGGCCCTTGCTGAATTACTTTGAACCTCTGTTTACG<br>TGGTTGAAAGACCAAAATAAAAACTCCTTTGTTGGGTGGAGTACTGACTGGTCCCCCTATGCGG<br>ACCAAAGCATCAAAGTGAGGATAAGCCTAAAATCAGCTCTTGGAGATAAAGCATATGAATGGAA<br>CGACAATGAAATGTACCTGTTCCGATCATCTGTTGCATATGCTATGAGGCAGTACTTTTTAAAA<br>GTAAAAAATCAGATGATTCTTTTTGGGGAGGAGGATGTGCGAGTGGCTAATTTGAAACCAAGAA<br>TCTCCTTTAATTTCTTTGTCACTGCACCTAAAAATGTGTCTGATATCATTCCTAGAACTGAAGT<br>TGAAAAGGCCATCAGGATGTCCCGGAGCCGTATCAATGATGCTTTCCGTCTGAATGACAACAGC<br>CTAGAGTTTCTGGGGATACAGCCAACACTTGGACCTCCTAACCAGCCCCCTGTTTCC
310	CAATCTACCATCGAAGAGCAGGTTAAATATTTCCTCGACAAGTTTAATGCTGAGGCTGAAGACC<br>TTGATTACCAATCAAGTCTGGCTAGCTGGAATTACAATACAAACATTACAGAGGAGAACGTACA<br>AAACATGAATAACGCAGGGGACAAGTGGAGCGCATTCCTTAAGGAACAAAGTACCCTTGCGCAA<br>ATGTATCCGCTGCAAGAGATTCAAAACCTGACGGTTAAGCTGCAACTTCAGGCCCTCCAACAAA<br>ATGGATCCTCAGTCTTGTCAGAAGACAAAAGCAAGCGACTGAACACCATCCTTAACACCATGTC<br>AACCATATATTCAACAGGTAAAGTTTGCAATCCGGATAACCCCCAAGAATGTTTGCTTCTTGAA<br>CCCGGTCTCAACGAAATTATGGCCAACAGTCTTGATTACAACGAGCGATTGTGGGCATGGGAAA<br>GTTGGAGGAGTGAGGTAGGCAAACAGTTGAGACCTCTTTATGAAGAGTACGTTGTCCTTAAAAA<br>TGAAATGGCTCGCGCGAATCATTATGAAGACTATGGTGACTACTGGAGGGGGGATTATGAGGTG<br>AACGGGGTGGACGGATACGATTACTCTAGGGGCCAGCTGATAGAGGATGTCGAGCACACCTTTG<br>AGGAGATTAAGCCGTTGTACGAACATTTGCACGCCTATGTCAGGGCTAAGCTCATGAACGCTTA<br>TCCGAGTTATATCTCCCCGATAGGATGCTTGCCTGCTCACTTGTTGGGCGATATGTGGGGACGC<br>TTTTGGACCAACTTGTATTCCCTTACGGTACCGTTCGGCCAGAAACCAAATATCGACGTGACAG<br>ACGCAATGGTGGATCAAGCATGGGATGCGCAACGAATCTTCAAGGAGGCAGAAAAATTTTTCGT<br>TTCAGTTGGACTCCCAAACATGACGCAGGGTTTCTGGGAGAACTCAATGTTGACAGATCCAGGT

	AATGTGCAGAAAGCGGTTTGCCTCCCTACTGCATGGGATCTTGGTAAAGGGGACTTCCGCATAC<br>TCATGTGTACGAAAGTAACTATGGACGACTTTCTTACTGCGCACCACGAGATGGGGCACATACA<br>ATACGATATGGCGTACGCAGCTCAACCTTTCCTTCTGCGGAACGGGGCGAATGAAGGATTTCAC<br>GAGGCAGTGGGTGAGATTATGTCCCTGTCAGCTGCCACTCCGAAACATCTGAAAAGCATCGGCC<br>TGTTGAGCCCAGACTTCCAAGAAGATAATGAGACCGAAATAAACTTCCTTCTGAAGCAAGCACT<br>GACTATTGTAGGTACCTTGCCCTTTACATACATGCTGGAGAAGTGGAGGTGGATGGTATTTAAG<br>GGGGAGATACCGAAAGATCAATGGATGAAAAAGTGGTGGGAAATGAAAAGGGAGATCGTTGGCG<br>TAGTTGAACCAGTACCGCATGATGAGACGTACTGCGATCCGGCTAGTCTGTTCCATGTCTCTAA<br>TGATTACTCTTTCATCCGCTACTACACCCGCACGCTGTATCAATTCCAGTTCCAAGAAGCTCTC<br>TGTCAGGCTGCCAAGCACGAAGGACCGCTGCACAAATGCGACATTAGCAATTCTACAGAGGCGG<br>GTCAGAAGTTGTTCAATATGCTTAGACTGGGGAAGAGCGAACCGTGGACGCTCGCTTTGGAGAA<br>CGTTGTTGGAGCTAAGAATATGAACGTCAGGCCCTTGCTGAATTACTTTGAACCTCTGTTTACG<br>TGGTTGAAAGACCAAAATAAAAACTCCTTTGTTGGGTGGAGTACTGACTGGTCCCCCTATGCGG<br>ACCAAAGCATCAAAGTGAGGATAAGCCTAAAATCAGCTCTTGGAGATAAAGCATATGAATGGAA<br>CGACAATGAAATGTACCTGTTCCGATCATCTGTTGCATATGCTATGAGGCAGTACTTTTTAAAA<br>GTAAAAAATCAGATGATTCTTTTTGGGGAGGAGGATGTGCGAGTGGCTAATTTGAAACCAAGAA<br>TCTCCTTTAATTTCTTTGTCACTGCACCTAAAAATGTGTCTGATATCATTCCTAGAACTGAAGT<br>TGAAAAGGCCATCAGGATGTCCCGGAGCCGTATCAATGATGCTTTCCGTCTGAATGACAACAGC<br>CTAGAGTTTCTGGGGATACAGCCAACACTTGGACCTCCTAACCAGCCCCCTGTTTCC
311	CAATCTACCATCGAAGAGCAGGCCAAAACATTCCTCGACTATTTTAATCACGAGGCTGAAGACC<br>TTTTCTACCAATCAAGTCTGGCTAGCTGGAATTACAATACAAACATTACAGAGGAGAACGTACA<br>AAACATGAATAACGCAGGGGACAAGGTTAGCGCATTCCTTAAGGAACAAAGTACCACTGCGCAA<br>ATGTATCCGCTGCAAGAGATTCAAAACCCAACGGTTAAGCTGCAACTTCAGGCCCTCCAACAAA<br>ATGGATCCTCAGTCTTGTCAGAAGACAAAAGCAAGCGACTGAACACCATCCTTAACACCATGTC<br>AACCATATATTCAACAGGTAAAGTTTGCAATCCGGATAACCCCCAAGAATGTTTGCTTCTTGAA<br>CCCGGTCTCAACGAAATTATGGCCAACAGTCTTGATTACAACGAGCGATTGTGGGCATGGGAAA<br>GTTGGAGGAGTGAGGTAGGCAAACAGTTGAGACCTCTTTATGAAGAGTACGTTGTCCTTAAAAA<br>TGAAATGGCTCGCGCGAATCATTATGAAGACTATGGTGACTACTGGAGGGGGGATTATGAGGTG<br>AACGGGGTGGACGGATACGATTACTCTAGGGGCCAGCTGATAGAGGATGTCGAGCACACCTTTG<br>AGGAGATTAAGCCGTTGTACGAACATTTGCACGCCTATGTCAGGGCTAAGCTCATGAACGCTTA<br>TCCGAGTTATATCTCCCCGATAGGATGCTTGCCTGCTCACTTGTTGGGCGATATGTGGGGACGC<br>TTTTGGACCAACTTGTATTCCCTTACGGTACCGTTCGGCCAGAAACCAAATATCGACGTGACAG<br>ACGCAATGGTGGATCAAGCATGGGATGCGCAACGAATCTTCAAGGAGGCAGAAAAATTTTTCGT<br>TTCAGTTGGACTCCCAAACATGACGCAGGGTTTCTGGGAGAACTCAATGTTGACAGATCCAGGT<br>AATGTGCAGAAAGCGGTTTGCCTCCCTACTGCATGGGATCTTGGTAAAGGGGACTTCCGCATAC<br>TCATGTGTACGAAAGTAACTATGGACGACTTTCTTACTGCGCACCACGAGATGGGGCACATACA<br>ATACGATATGGCGTACGCAGCTCAACCTTTCCTTCTGCGGAACGGGGCGAATGAAGGATTTCAC<br>GAGGCAGTGGGTGAGATTATGTCCCTGTCAGCTGCCACTCCGAAACATCTGAAAAGCATCGGCC<br>TGTTGAGCCCAGACTTCCAAGAAGATAATGAGACCGAAATAAACTTCCTTCTGAAGCAAGCACT<br>GACTATTGTAGGTACCTTGCCCTTTACATACATGCTGGAGAAGTGGAGGTGGATGGTATTTAAG<br>GGGGAGATACCGAAAGATCAATGGATGAAAAAGTGGTGGGAAATGAAAAGGGAGATCGTTGGCG<br>TAGTTGAACCAGTACCGCATGATGAGACGTACTGCGATCCGGCTAGTCTGTTCCATGTCTCTAA<br>TGATTACTCTTTCATCCGCTACTACACCCGCACGCTGTATCAATTCCAGTTCCAAGAAGCTCTC<br>TGTCAGGCTGCCAAGCACGAAGGACCGCTGCACAAATGCGACATTAGCAATTCTACAGAGGCGG<br>GTCAGAAGTTGTTCAATATGCTTAGACTGGGGAAGAGCGAACCGTGGACGCTCGCTTTGGAGAA<br>CGTTGTTGGAGCTAAGAATATGAACGTCAGGCCCTTGCTGAATTACTTTGAACCTCTGTTTACG<br>TGGTTGAAAGACCAAAATAAAAACTCCTTTGTTGGGTGGAGTACTGACTGGTCCCCCTATGCGG

	ACCAAAGCATCAAAGTGAGGATAAGCCTAAAATCAGCTCTTGGAGATAAAGCATATGAATGGAA CGACAATGAAATGTACCTGTTCCGATCATCTGTTGCATATGCTATGAGGCAGTACTTTTTAAAA GTAAAAAATCAGATGATTCTTTTTGGGGAGGAGGATGTGCGAGTGGCTAATTTGAAACCAAGAA TCTCCTTTAATTTCTTTGTCACTGCACCTAAAAATGTGTCTGATATCATTCCTAGAACTGAAGT TGAAAAGGCCATCAGGATGTCCCGGAGCCGTATCAATGATGCTTTCCGTCTGAATGACAACAGC CTAGAGTTTCTGGGGATACAGCCAACACTTGGACCTCCTAACCAGCCCCCTGTTTCC
312	CAATCTACCATCGAAGAGCAGGCCAAATATTTCCTCGACAAGTTTAATGCTGAGGCTGAAGACC TTTTCTACCAATCAAGTCTGGCTAGCTGGAATTACAATACAAACATTACAGAGGAGAACGTACA AAACATGAATAACGCAGGGGACAAGTGGAGCGCATTCCTTAAGGAACAAAGTACCCTTGCGCAA ATGTATCCGCTGCAAGAGATTCAACAACTGACGGTTAAGCTGCAACTTCAGGCCCTCCAACAAA ATGGATCCTCAGTCTTGTCAGAAGACAAAAGCAAGCGACTGAACACCATCCTTAACACCATGTC AACCATATATTCAACAGGTAAAGTTTGCAATCCGGATAACCCCCAAGAATGTTTGCTTCTTGAA CCCGGTCTCAACGAAATTATGGCCAACAGTCTTGATTACAACGAGCGATTGTGGGCATGGGAAA GTTGGAGGAGTGAGGTAGGCAAACAGTTGAGACCTCTTTATGAAGAGTACGTTGTCCTTAAAAA TGAAATGGCTCGCGCGAATCATTATGAAGACTATGGTGACTACTGGAGGGGGGATTATGAGGTG AACGGGGTGGACGGATACGATTACTCTAGGGGCCAGCTGATAGAGGATGTCGAGCACACCTTTG AGGAGATTAAGCCGTTGTACGAACATTTGCACGCCTATGTCAGGGCTAAGCTCATGAACGCTTA TCCGAGTTATATCTCCCCGATAGGATGCTTGCCTGCTCACTTGTTGGGCGATATGTGGGGACGC TTTTGGACCAACTTGTATTCCCTTACGGTACCGTTCGGCCAGAAACCAAATATCGACGTGACAG ACGCAATGGTGGATCAAGCATGGGATGCGCAACGAATCTTCAAGGAGGCAGAAAAATTTTTCGT TTCAGTTGGACTCCCAAACATGACGCAGGGTTTCTGGGAGAACTCAATGTTGACAGATCCAGGT AATGTGCAGAAAGCGGTTTGCCTCCCTACTGCATGGGATCTTGGTAAAGGGGACTTCCGCATAC TCATGTGTACGAAAGTAACTATGGACGACTTTCTTACTGCGCACCACGAGATGGGGCACATACA ATACGATATGGCGTACGCAGCTCAACCTTTCCTTCTGCGGAACGGGGCGAATGAAGGATTTCAC GAGGCAGTGGGTGAGATTATGTCCCTGTCAGCTGCCACTCCGAAACATCTGAAAAGCATCGGCC TGTTGAGCCCAGACTTCCAAGAAGATAATGAGACCGAAATAAACTTCCTTCTGAAGCAAGCACT GACTATTGTAGGTACCTTGCCCTTTACATACATGCTGGAGAAGTGGAGGTGGATGGTATTTAAG GGGGAGATACCGAAAGATCAATGGATGAAAAAGTGGTGGGAAATGAAAAGGGAGATCGTTGGCG TAGTTGAACCAGTACCGCATGATGAGACGTACTGCGATCCGGCTAGTCTGTTCCATGTCTCTAA TGATTACTCTTTCATCCGCTACTACACCCGCACGCTGTATCAATTCCAGTTCCAAGAAGCTCTC TGTCAGGCTGCCAAGCACGAAGGACCGCTGCACAAATGCGACATTAGCAATTCTACAGAGGCGG GTCAGAAGTTGTTCAATATGCTTAGACTGGGGAAGAGCGAACCGTGGACGCTCGCTTTGGAGAA CGTTGTTGGAGCTAAGAATATGAACGTCAGGCCCTTGCTGAATTACTTTGAACCTCTGTTTACG TGGTTGAAAGACCAAAATAAAAACTCCTTTGTTGGGTGGAGTACTGACTGGTCCCCCTATGCGG ACCAAAGCATCAAAGTGAGGATAAGCCTAAAATCAGCTCTTGGAGATAAAGCATATGAATGGAA CGACAATGAAATGTACCTGTTCCGATCATCTGTTGCATATGCTATGAGGCAGTACTTTTTAAAA GTAAAAAATCAGATGATTCTTTTTGGGGAGGAGGATGTGCGAGTGGCTAATTTGAAACCAAGAA TCTCCTTTAATTTCTTTGTCACTGCACCTAAAAATGTGTCTGATATCATTCCTAGAACTGAAGT TGAAAAGGCCATCAGGATGTCCCGGAGCCGTATCAATGATGCTTTCCGTCTGAATGACAACAGC CTAGAGTTTCTGGGGATACAGCCAACACTTGGACCTCCTAACCAGCCCCCTGTTTCC
313	CAATCTACCATCGAAGAGCAGGCCAAAACATTCCTCGACTTCTTTGATAGCCAGGCTGAAGACC TTTTCTACCAATCAAGTCTGGCTAGCTGGAATTACAATACAAACATTACAGAGGAGAACGTACA AAACATGAATAACGCAGGGGACAAGTGGAGCGCATTCCTTAAGGAACAAAGTACCCTTGCGCAA ATGTATCCGCTGCAAGAGATTCAAAACCTGACGGTTAAGCTGCAACTTCAGGCCCTCCAACAAA ATGGATCCTCAGTCTTGTCAGAAGACAAAAGCAAGCGACTGAACACCATCCTTAACACCATGTC AACCATATATTCAACAGGTAAAGTTTGCAATCCGGATAACCCCCAAGAATGTTTGCTTCTTGAA CCCGGTCTCAACGAAATTATGGCCAACAGTCTTGATTACAACGAGCGATTGTGGGCATGGGAAA

	GTTGGAGGAGTGAGGTAGGCAAACAGTTGAGACCTCTTTATGAAGAGTACGTTGTCCTTAAAAA<br>TGAAATGGCTCGCGCGAATCATTATGAAGACTATGGTGACTACTGGAGGGGGGATTATGAGGTG<br>AACGGGGTGGACGGATACGATTACTCTAGGGGCCAGCTGATAGAGGATGTCGAGCACACCTTTG<br>AGGAGATTAAGCCGTTGTACGAACATTTGCACGCCTATGTCAGGGCTAAGCTCATGAACGCTTA<br>TCCGAGTTATATCTCCCCGATAGGATGCTTGCCTGCTCACTTGTTGGGCGATATGTGGGGACGC<br>TTTTGGACCAACTTGTATTCCCTTACGGTACCGTTCGGCCAGAAACCAAATATCGACGTGACAG<br>ACGCAATGGTGGATCAAGCATGGGATGCGCAACGAATCTTCAAGGAGGCAGAAAAATTTTTCGT<br>TTCAGTTGGACTCCCAAACATGACGCAGGGTTTCTGGGAGAACTCAATGTTGACAGATCCAGGT<br>AATGTGCAGAAAGCGGTTTGCCTCCCTACTGCATGGGATCTTGGTAAAGGGGACTTCCGCATAC<br>TCATGTGTACGAAAGTAACTATGGACGACTTTCTTACTGCGCACCACGAGATGGGGCACATACA<br>ATACGATATGGCGTACGCAGCTCAACCTTTCCTTCTGCGGAACGGGGCGAATGAAGGATTTCAC<br>GAGGCAGTGGGTGAGATTATGTCCCTGTCAGCTGCCACTCCGAAACATCTGAAAAGCATCGGCC<br>TGTTGAGCCCAGACTTCCAAGAAGATAATGAGACCGAAATAAACTTCCTTCTGAAGCAAGCACT<br>GACTATTGTAGGTACCTTGCCCTTTACATACATGCTGGAGAAGTGGAGGTGGATGGTATTTAAG<br>GGGGAGATACCGAAAGATCAATGGATGAAAAAGTGGTGGGAAATGAAAAGGGAGATCGTTGGCG<br>TAGTTGAACCAGTACCGCATGATGAGACGTACTGCGATCCGGCTAGTCTGTTCCATGTCTCTAA<br>TGATTACTCTTTCATCCGCTACTACACCCGCACGCTGTATCAATTCCAGTTCCAAGAAGCTCTC<br>TGTCAGGCTGCCAAGCACGAAGGACCGCTGCACAAATGCGACATTAGCAATTCTACAGAGGCGG<br>GTCAGAAGTTGTTCAATATGCTTAGACTGGGGAAGAGCGAACCGTGGACGCTCGCTTTGGAGAA<br>CGTTGTTGGAGCTAAGAATATGAACGTCAGGCCCTTGCTGAATTACTTTGAACCTCTGTTTACG<br>TGGTTGAAAGACCAAAATAAAAACTCCTTTGTTGGGTGGAGTACTGACTGGTCCCCCTATGCGG<br>ACCAAAGCATCAAAGTGAGGATAAGCCTAAAATCAGCTCTTGGAGATAAAGCATATGAATGGAA<br>CGACAATGAAATGTACCTGTTCCGATCATCTGTTGCATATGCTATGAGGCAGTACTTTTTAAAA<br>GTAAAAAATCAGATGATTCTTTTTGGGGAGGAGGATGTGCGAGTGGCTAATTTGAAACCAAGAA<br>TCTCCTTTAATTTCTTTGTCACTGCACCTAAAAATGTGTCTGATATCATTCCTAGAACTGAAGT<br>TGAAAAGGCCATCAGGATGTCCCGGAGCCGTATCAATGATGCTTTCCGTCTGAATGACAACAGC<br>CTAGAGTTTCTGGGGATACAGCCAACACTTGGACCTCCTAACCAGCCCCCTGTTTCC
353	CAATCTACCATCGAAGAGCAGGCCAAAGCATTCCTCGACTTCTTTGATAGCCAGGCTGAAGACC<br>TTTTCTACCAATCAAGTCTGGCTAGCTGGAATTACAATACAAACATTACAGAGGAGAACGTACA<br>AGACATGAATAACGCAGGGGACAGGTGGAGCGCATTCCTTAAGGAACAAAGTACCCCTGCGCAA<br>ATGTATCCGCTGCAAGAGATTCAAAACCTGACGGTTAAGCTGCAACTTCAGGCCCTCCAACAAA<br>ATGGATCCTCAGTCTTGTCAGAAGACAAAAGCAAGCGACTGAACACCATCCTTAACACCATGTC<br>AACCATATATTCAACAGGTAAAGTTTGCAATCCGGATAACCCCCAAGAATGTTTGCTTCTTGAA<br>CCCGGTCTCAACGAAATTATGGCCAACAGTCTTGATTACAACGAGCGATTGTGGGCATGGGAAA<br>GTTGGAGGAGTGAGGTAGGCAAACAGTTGAGACCTCTTTATGAAGAGTACGTTGTCCTTAAAAA<br>TGAAATGGCTCGCGCGAATCATTATGAAGACTATGGTGACTACTGGAGGGGGGATTATGAGGTG<br>AACGGGGTGGACGGATACGATTACTCTAGGGGCCAGCTGATAGAGGATGTCGAGCACACCTTTG<br>AGGAGATTAAGCCGTTGTACGAACATTTGCACGCCTATGTCAGGGCTAAGCTCATGAACGCTTA<br>TCCGAGTTATATCTCCCCGATAGGATGCTTGCCTGCTCACTTGTTGGGCGATATGTGGGGACGC<br>TTTTGGACCAACTTGTATTCCCTTACGGTACCGTTCGGCCAGAAACCAAATATCGACGTGACAG<br>ACGCAATGGTGGATCAAGCATGGGATGCGCAACGAATCTTCAAGGAGGCAGAAAAATTTTTCGT<br>TTCAGTTGGACTCCCAAACATGACGCAGGGTTTCTGGGAGAACTCAATGTTGACAGATCCAGGT<br>AATGTGCAGAAAGCGGTTTGCCTCCCTACTGCATGGGATCTTGGTAAAGGGGACTTCCGCATAC<br>TCATGTGTACGAAAGTAACTATGGACGACTTTCTTACTGCGCACCACGAGATGGGGCACATACA<br>ATACGATATGGCGTACGCAGCTCAACCTTTCCTTCTGCGGAACGGGGCGAATGAAGGATTTCAC<br>GAGGCAGTGGGTGAGATTATGTCCCTGTCAGCTGCCACTCCGAAACATCTGAAAAGCATCGGCC<br>TGTTGAGCCCAGACTTCCAAGAAGATAATGAGACCGAAATAAACTTCCTTCTGAAGCAAGCACT

	GACTATTGTAGGTACCTTGCCCTTTACATACATGCTGGAGAAGTGGAGGTGGATGGTATTTAAG<br>GGGGAGATACCGAAAGATCAATGGATGAAAAAGTGGTGGGAAATGAAAAGGGAGATCGTTGGCG<br>TAGTTGAACCAGTACCGCATGATGAGACGTACTGCGATCCGGCTAGTCTGTTCCATGTCTCTAA<br>TGATTACTCTTTCATCCGCTACTACACCCGCACGCTGTATCAATTCCAGTTCCAAGAAGCTCTC<br>TGTCAGGCTGCCAAGCACGAAGGACCGCTGCACAAATGCGACATTAGCAATTCTACAGAGGCGG<br>GTCAGAAGTTGTTCAATATGCTTAGACTGGGGAAGAGCGAACCGTGGACGCTCGCTTTGGAGAA<br>CGTTGTTGGAGCTAAGAATATGAACGTCAGGCCCTTGCTGAATTACTTTGAACCTCTGTTTACG<br>TGGTTGAAAGACCAAAATAAAAACTCCTTTGTTGGGTGGAGTACTGACTGGTCCCCCTATGCGG<br>ACCAAAGCATCAAAGTGAGGATAAGCCTAAAATCAGCTCTTGGAGATAAAGCATATGAATGGAA<br>CGACAATGAAATGTACCTGTTCCGATCATCTGTTGCATATGCTATGAGGCAGTACTTTTTAAAA<br>GTAAAAAATCAGATGATTCTTTTTGGGGAGGAGGATGTGCGAGTGGCTAATTTGAAACCAAGAA<br>TCTCCTTTAATTTCTTTGTCACTGCACCTAAAAATGTGTCTGATATCATTCCTAGAACTGAAGT<br>TGAAAAGGCCATCAGGATGTCCCGGAGCCGTATCAATGATGCTTTCCGTCTGAATGACAACAGC<br>CTAGAGTTTCTGGGGATACAGCCAACACTTGGACCTCCTAACCAGCCCCCTGTTTCC
354	CAATCTACCATCGAAGAGCAGGCCAAAACATTCCTCGACTTCTTTGATGCCCAGGCTGAAGACC<br>TTTTCTACCAATCAAGTCTGGCTAGCTGGGATTACAGTACAAGCATTACAGAGGGGAACGTGCA<br>AAACATGAATGACGCAGGGGACAAGTGGAGCGCATTCCTTAAGGAGCAAAGTACCCTTGCGCAA<br>ATGTATCCGCTGCAAGAGATTCAAAACCTGACGGTTAAGCTGCAACTTCAGGCCCTCCAGCAAA<br>ATGGATCCTCAGTCTTGTCAGAAGACAAAAGCAAGCGACTGAACACCATCCTTAACACCATGTC<br>AACCATATATTCAACAGGTAAAGTTTGCAATCCGGATAACCCCCAAGAATGTTTGCTTCTTGAA<br>CCCGGTCTCAACGAAATTATGGCCAACAGTCTTGATTACAACGAGCGATTGTGGGCATGGGAAA<br>GTTGGAGGAGTGAGGTAGGCAAACAGTTGAGACCTCTTTATGAAGAGTACGTTGTCCTTAAAAA<br>TGAAATGGCTCGCGCGAATCATTATGAAGACTATGGTGACTACTGGAGGGGGGATTATGAGGTG<br>AACGGGGTGGACGGATACGATTACTCTAGGGGCCAGCTGATAGAGGATGTCGAGCACACCTTTG<br>AGGAGATTAAGCCGTTGTACGAACATTTGCACGCCTATGTCAGGGCTAAGCTCATGAACGCTTA<br>TCCGAGTTATATCTCCCCGATAGGATGCTTGCCTGCTCACTTGTTGGGCGATATGTGGGGACGC<br>TTTTGGACCAACTTGTATTCCCTTACGGTACCGTTCGGCCAGAAACCAAATATCGACGTGACAG<br>ACGCAATGGTGGATCAAGCATGGGATGCGCAACGAATCTTCAAGGAGGCAGAAAAATTTTTCGT<br>TTCAGTTGGACTCCCAAACATGACGCAGGGTTTCTGGGAGAACTCAATGTTGACAGATCCAGGT<br>AATGTGCAGAAAGCGGTTTGCCTCCCTACTGCATGGGATCTTGGTAAAGGGGACTTCCGCATAC<br>TCATGTGTACGAAAGTAACTATGGACGACTTTCTTACTGCGCACCACGAGATGGGGCACATACA<br>ATACGATATGGCGTACGCAGCTCAACCTTTCCTTCTGCGGAACGGGGCGAATGAAGGATTTCAC<br>GAGGCAGTGGGTGAGATTATGTCCCTGTCAGCTGCCACTCCGAAACATCTGAAAAGCATCGGCC<br>TGTTGAGCCCAGACTTCCAAGAAGATAATGAGACCGAAATAAACTTCCTTCTGAAGCAAGCACT<br>GACTATTGTAGGTACCTTGCCCTTTACATACATGCTGGAGAAGTGGAGGTGGATGGTATTTAAG<br>GGGGAGATACCGAAAGATCAATGGATGAAAAAGTGGTGGGAAATGAAAAGGGAGATCGTTGGCG<br>TAGTTGAACCAGTACCGCATGATGAGACGTACTGCGATCCGGCTAGTCTGTTCCATGTCTCTAA<br>TGATTACTCTTTCATCCGCTACTACACCCGCACGCTGTATCAATTCCAGTTCCAAGAAGCTCTC<br>TGTCAGGCTGCCAAGCACGAAGGACCGCTGCACAAATGCGACATTAGCAATTCTACAGAGGCGG<br>GTCAGAAGTTGTTCAATATGCTTAGACTGGGGAAGAGCGAACCGTGGACGCTCGCTTTGGAGAA<br>CGTTGTTGGAGCTAAGAATATGAACGTCAGGCCCTTGCTGAATTACTTTGAACCTCTGTTTACG<br>TGGTTGAAAGACCAAAATAAAAACTCCTTTGTTGGGTGGAGTACTGACTGGTCCCCCTATGCGG<br>ACCAAAGCATCAAAGTGAGGATAAGCCTAAAATCAGCTCTTGGAGATAAAGCATATGAATGGAA<br>CGACAATGAAATGTACCTGTTCCGATCATCTGTTGCATATGCTATGAGGCAGTACTTTTTAAAA<br>GTAAAAAATCAGATGATTCTTTTTGGGGAGGAGGATGTGCGAGTGGCTAATTTGAAACCAAGAA<br>TCTCCTTTAATTTCTTTGTCACTGCACCTAAAAATGTGTCTGATATCATTCCTAGAACTGAAGT

	TGAAAAGGCCATCAGGATGTCCCGGAGCCGTATCAATGATGCTTTCCGTCTGAATGACAACAGC<br>CTAGAGTTTCTGGGGATACAGCCAACACTTGGACCTCCTAACCAGCCCCCTGTTTCC
355	CAACCAACCATCGAAGAGCAGGCCAAAACATTCCTCGACAAGTTTAATCACGAGGCTGAAGACC<br>TTTTCTACTTGTCAAGTCTGGCTAGCTGGAATTACAATACAAACATTACAGAGGAGAACGTACA<br>AAACATGAATAACGCAGGGGACAAGTGGAGCGCATTCCTTAAGGAACAAAGTACCACTGCGCAA<br>ATGTATCCGCTGCAAGAGATTCAACAGCTGACGGTTAAGCTGCAACTTCAGGCCCTCCAACAAA<br>ATGGATCCTCAGTCTTGTCAGAAGACAAAAGCAAGCGACTGAACACCATCCTTAACACCATGTC<br>AACCATATATTCAACAGGTAAAGTTTGCAATCCGGATAACCCCCAAGAATGTTTGCTTCTTGAA<br>CCCGGTCTCAACGAAATTATGGCCAACAGTCTTGATTACAACGAGCGATTGTGGGCATGGGAAA<br>GTTGGAGGAGTGAGGTAGGCAAACAGTTGAGACCTCTTTATGAAGAGTACGTTGTCCTTAAAAA<br>TGAAATGGCTCGCGCGAATCATTATGAAGACTATGGTGACTACTGGAGGGGGGATTATGAGGTG<br>AACGGGGTGGACGGATACGATTACTCTAGGGGCCAGCTGATAGAGGATGTCGAGCACACCTTTG<br>AGGAGATTAAGCCGTTGTACGAACATTTGCACGCCTATGTCAGGGCTAAGCTCATGAACGCTTA<br>TCCGAGTTATATCTCCCCGATAGGATGCTTGCCTGCTCACTTGTTGGGCGATATGTGGGGACGC<br>TTTTGGACCAACTTGTATTCCCTTACGGTACCGTTCGGCCAGAAACCAAATATCGACGTGACAG<br>ACGCAATGGTGGATCAAGCATGGGATGCGCAACGAATCTTCAAGGAGGCAGAAAAATTTTTCGT<br>TTCAGTTGGACTCCCAAACATGACGCAGGGTTTCTGGGAGAACTCAATGTTGACAGATCCAGGT<br>AATGTGCAGAAAGCGGTTTGCCTCCCTACTGCATGGGATCTTGGTAAAGGGGACTTCCGCATAC<br>TCATGTGTACGAAAGTAACTATGGACGACTTTCTTACTGCGCACCACGAGATGGGGCACATACA<br>ATACGATATGGCGTACGCAGCTCAACCTTTCCTTCTGCGGAACGGGGCGAATGAAGGATTTCAC<br>GAGGCAGTGGGTGAGATTATGTCCCTGTCAGCTGCCACTCCGAAACATCTGAAAAGCATCGGCC<br>TGTTGAGCCCAGACTTCCAAGAAGATAATGAGACCGAAATAAACTTCCTTCTGAAGCAAGCACT<br>GACTATTGTAGGTACCTTGCCCTTTACATACATGCTGGAGAAGTGGAGGTGGATGGTATTTAAG<br>GGGGAGATACCGAAAGATCAATGGATGAAAAAGTGGTGGGAAATGAAAAGGGAGATCGTTGGCG<br>TAGTTGAACCAGTACCGCATGATGAGACGTACTGCGATCCGGCTAGTCTGTTCCATGTCTCTAA<br>TGATTACTCTTTCATCCGCTACTACACCCGCACGCTGTATCAATTCCAGTTCCAAGAAGCTCTC<br>TGTCAGGCTGCCAAGCACGAAGGACCGCTGCACAAATGCGACATTAGCAATTCTACAGAGGCGG<br>GTCAGAAGTTGTTCAATATGCTTAGACTGGGGAAGAGCGAACCGTGGACGCTCGCTTTGGAGAA<br>CGTTGTTGGAGCTAAGAATATGAACGTCAGGCCCTTGCTGAATTACTTTGAACCTCTGTTTACG<br>TGGTTGAAAGACCAAAATAAAAACTCCTTTGTTGGGTGGAGTACTGACTGGTCCCCCTATGCGG<br>ACCAAAGCATCAAAGTGAGGATAAGCCTAAAATCAGCTCTTGGAGATAAAGCATATGAATGGAA<br>CGACAATGAAATGTACCTGTTCCGATCATCTGTTGCATATGCTATGAGGCAGTACTTTTTAAAA<br>GTAAAAAATCAGATGATTCTTTTTGGGGAGGAGGATGTGCGAGTGGCTAATTTGAAACCAAGAA<br>TCTCCTTTAATTTCTTTGTCACTGCACCTAAAAATGTGTCTGATATCATTCCTAGAACTGAAGT<br>TGAAAAGGCCATCAGGATGTCCCGGAGCCGTATCAATGATGCTTTCCGTCTGAATGACAACAGC<br>CTAGAGTTTCTGGGGATACAGCCAACACTTGGACCTCCTAACCAGCCCCCTGTTTCC
373	CGATCTACCATCGAAGAGCAGGCCAAAACATTCCTCGACTTCTTTGATAGCCAGGCTGAAGACC<br>TTTTCTACCAATCAAGTCTGGCAAGCTGGAATTACAACACAAACATTACAGAGGAGAACGTACA<br>AAACATGAATAACGCAGGGGACAAGCGGAGCGCATTCCTTAAGGAACGAAGTACCCTTGCGCAG<br>ATGTATCCGCTGCAAGAGATTCAAAACCTGACGGTTAAGCTGCAACTTCAGGCCCTCCAACAAA<br>ATGGATCCTCAGTCTTGTCAGAAGACAAAAGCAAGCGACTGAACACCATCCTTAACACCATGTC<br>AACCATATATTCAACAGGTAAAGTTTGCAATCCGGATAACCCCCAAGAATGTTTGCTTCTTGAA<br>CCCGGTCTCAACGAAATTATGGCCAACAGTCTTGATTACAACGAGCGATTGTGGGCATGGGAAA<br>GTTGGAGGAGTGAGGTAGGCAAACAGTTGAGACCTCTTTATGAAGAGTACGTTGTCCTTAAAAA<br>TGAAATGGCTCGCGCGAATCATTATGAAGACTATGGTGACTACTGGAGGGGGGATTATGAGGTG<br>AACGGGGTGGACGGATACGATTACTCTAGGGGCCAGCTGATAGAGGATGTCGAGCACACCTTTG<br>AGGAGATTAAGCCGTTGTACGAACATTTGCACGCCTATGTCAGGGCTAAGCTCATGAACGCTTA

	TCCGAGTTATATCTCCCCGATAGGATGCTTGCCTGCTCACTTGTTGGGCGATATGTGGGGACGC<br>TTTTGGACCAACTTGTATTCCCTTACGGTACCGTTCGGCCAGAAACCAAATATCGACGTGACAG<br>ACGCAATGGTGGATCAAGCATGGGATGCGCAACGAATCTTCAAGGAGGCAGAAAAATTTTTCGT<br>TTCAGTTGGACTCCCAAACATGACGCAGGGTTTCTGGGAGAACTCAATGTTGACAGATCCAGGT<br>AATGTGCAGAAAGCGGTTTGCCTCCCTACTGCATGGGATCTTGGTAAAGGGGACTTCCGCATAC<br>TCATGTGTACGAAAGTAACTATGGACGACTTTCTTACTGCGCACCACGAGATGGGGCACATACA<br>ATACGATATGGCGTACGCAGCTCAACCTTTCCTTCTGCGGAACGGGGCGAATGAAGGATTTCAC<br>GAGGCAGTGGGTGAGATTATGTCCCTGTCAGCTGCCACTCCGAAACATCTGAAAAGCATCGGCC<br>TGTTGAGCCCAGACTTCCAAGAAGATAATGAGACCGAAATAAACTTCCTTCTGAAGCAAGCACT<br>GACTATTGTAGGTACCTTGCCCTTTACATACATGCTGGAGAAGTGGAGGTGGATGGTATTTAAG<br>GGGGAGATACCGAAAGATCAATGGATGAAAAAGTGGTGGGAAATGAAAAGGGAGATCGTTGGCG<br>TAGTTGAACCAGTACCGCATGATGAGACGTACTGCGATCCGGCTAGTCTGTTCCATGTCTCTAA<br>TGATTACTCTTTCATCCGCTACTACACCCGCACGCTGTATCAATTCCAGTTCCAAGAAGCTCTC<br>TGTCAGGCTGCCAAGCACGAAGGACCGCTGCACAAATGCGACATTAGCAATTCTACAGAGGCGG<br>GTCAGAAGTTGTTCAATATGCTTAGACTGGGGAAGAGCGAACCGTGGACGCTCGCTTTGGAGAA<br>CGTTGTTGGAGCTAAGAATATGAACGTCAGGCCCTTGCTGAATTACTTTGAACCTCTGTTTACG<br>TGGTTGAAAGACCAAAATAAAAACTCCTTTGTTGGGTGGAGTACTGACTGGTCCCCCTATGCGG<br>ACCAAAGCATCAAAGTGAGGATAAGCCTAAAATCAGCTCTTGGAGATAAAGCATATGAATGGAA<br>CGACAATGAAATGTACCTGTTCCGATCATCTGTTGCATATGCTATGAGGCAGTACTTTTTAAAA<br>GTAAAAAATCAGATGATTCTTTTTGGGGAGGAGGATGTGCGAGTGGCTAATTTGAAACCAAGAA<br>TCTCCTTTAATTTCTTTGTCACTGCACCTAAAAATGTGTCTGATATCATTCCTAGAACTGAAGT<br>TGAAAAGGCCATCAGGATGTCCCGGAGCCGTATCAATGATGCTTTCCGTCTGAATGACAACAGC<br>CTAGAGTTTCTGGGGATACAGCCAACACTTGGACCTCCTAACCAGCCCCCTGTTTCC
375	CAACCTACCATCGAAGAGCAGGCCAAAACATTCCTCGACAAGTTTAGTGTCGAGGCTGAAGACC<br>TTCTCTACCAATCAAGTCTGGCTAGCTGGGATTACAACACAAACATTACAGAGGAGAACGTACA<br>AAACATGAATAACGCAGGGGACAAATGGAGCGCATTCCTCAAGGAACAAAGTACCCTTGCGCAA<br>ATGTATCCGCTGCAAGAGATTCAAAACCTGACGGTTAAGCTGCAACTTCAGGCCCCCCAACAAA<br>ATGGATCCTCAGTCTTGTCAGAAGACAAAAGCAAGCGACTGAACACCATCCTTAACACCATGTC<br>AACCATATATTCAACAGGTAAAGTTTGCAATCCGGATAACCCCCAAGAATGTTTGCTTCTTGAA<br>CCCGGTCTCAACGAAATTATGGCCAACAGTCTTGATTACAACGAGCGATTGTGGGCATGGGAAA<br>GTTGGAGGAGTGAGGTAGGCAAACAGTTGAGACCTCTTTATGAAGAGTACGTTGTCCTTAAAAA<br>TGAAATGGCTCGCGCGAATCATTATGAAGACTATGGTGACTACTGGAGGGGGGATTATGAGGTG<br>AACGGGGTGGACGGATACGATTACTCTAGGGGCCAGCTGATAGAGGATGTCGAGCACACCTTTG<br>AGGAGATTAAGCCGTTGTACGAACATTTGCACGCCTATGTCAGGGCTAAGCTCATGAACGCTTA<br>TCCGAGTTATATCTCCCCGATAGGATGCTTGCCTGCTCACTTGTTGGGCGATATGTGGGGACGC<br>TTTTGGACCAACTTGTATTCCCTTACGGTACCGTTCGGCCAGAAACCAAATATCGACGTGACAG<br>ACGCAATGGTGGATCAAGCATGGGATGCGCAACGAATCTTCAAGGAGGCAGAAAAATTTTTCGT<br>TTCAGTTGGACTCCCAAACATGACGCAGGGTTTCTGGGAGAACTCAATGTTGACAGATCCAGGT<br>AATGTGCAGAAAGCGGTTTGCCTCCCTACTGCATGGGATCTTGGTAAAGGGGACTTCCGCATAC<br>TCATGTGTACGAAAGTAACTATGGACGACTTTCTTACTGCGCACCACGAGATGGGGCACATACA<br>ATACGATATGGCGTACGCAGCTCAACCTTTCCTTCTGCGGAACGGGGCGAATGAAGGATTTCAC<br>GAGGCAGTGGGTGAGATTATGTCCCTGTCAGCTGCCACTCCGAAACATCTGAAAAGCATCGGCC<br>TGTTGAGCCCAGACTTCCAAGAAGATAATGAGACCGAAATAAACTTCCTTCTGAAGCAAGCACT<br>GACTATTGTAGGTACCTTGCCCTTTACATACATGCTGGAGAAGTGGAGGTGGATGGTATTTAAG<br>GGGGAGATACCGAAAGATCAATGGATGAAAAAGTGGTGGGAAATGAAAAGGGAGATCGTTGGCG<br>TAGTTGAACCAGTACCGCATGATGAGACGTACTGCGATCCGGCTAGTCTGTTCCATGTCTCTAA<br>TGATTACTCTTTCATCCGCTACTACACCCGCACGCTGTATCAATTCCAGTTCCAAGAAGCTCTC

	TGTCAGGCTGCCAAGCACGAAGGACCGCTGCACAAATGCGACATTAGCAATTCTACAGAGGCGG<br>GTCAGAAGTTGTTCAATATGCTTAGACTGGGGAAGAGCGAACCGTGGACGCTCGCTTTGGAGAA<br>CGTTGTTGGAGCTAAGAATATGAACGTCAGGCCCTTGCTGAATTACTTTGAACCTCTGTTTACG<br>TGGTTGAAAGACCAAAATAAAAACTCCTTTGTTGGGTGGAGTACTGACTGGTCCCCCTATGCGG<br>ACCAAAGCATCAAAGTGAGGATAAGCCTAAAATCAGCTCTTGGAGATAAAGCATATGAATGGAA<br>CGACAATGAAATGTACCTGTTCCGATCATCTGTTGCATATGCTATGAGGCAGTACTTTTTAAAA<br>GTAAAAAATCAGATGATTCTTTTTGGGGAGGAGGATGTGCGAGTGGCTAATTTGAAACCAAGAA<br>TCTCCTTTAATTTCTTTGTCACTGCACCTAAAAATGTGTCTGATATCATTCCTAGAACTGAAGT<br>TGAAAAGGCCATCAGGATGTCCCGGAGCCGTATCAATGATGCTTTCCGTCTGAATGACAACAGC<br>CTAGAGTTTCTGGGGATACAGCCAACACTTGGACCTCCTAACCAGCCCCCTGTTTCC
